# Neuronal activity in the monkey prefrontal cortex during a duration discrimination task with visual and auditory cues

**DOI:** 10.1038/s41598-021-97094-w

**Published:** 2021-09-01

**Authors:** Atsushi Chiba, Kazunori Morita, Ken-ichi Oshio, Masahiko Inase

**Affiliations:** 1 Department of Physiology, Kindai University Faculty of Medicine, Osaka-Sayama, Osaka 589-8511 Japan; 2 Department of Physiology, School of Medicine, Iwate Medical University, Yahaba, Iwate 028-3694 Japan

**Keywords:** Decision, Perception

## Abstract

To investigate neuronal processing involved in the integration of auditory and visual signals for time perception, we examined neuronal activity in prefrontal cortex (PFC) of macaque monkeys during a duration discrimination task with auditory and visual cues. In the task, two cues were consecutively presented for different durations between 0.2 and 1.8 s. Each cue was either auditory or visual and was followed by a delay period. After the second delay, subjects indicated whether the first or the second cue was longer. Cue- and delay-responsive neurons were found in PFC. Cue-responsive neurons mostly responded to either the auditory or the visual cue, and to either the first or the second cue. The neurons responsive to the first delay showed activity that changed depending on the first cue duration and were mostly sensitive to cue modality. The neurons responsive to the second delay exhibited activity that represented which cue, the first or second cue, was presented longer. Nearly half of this activity representing order-based duration was sensitive to cue modality. These results suggest that temporal information with visual and auditory signals was separately processed in PFC in the early stage of duration discrimination and integrated for the final decision.

## Introduction

Integration of multisensory information is crucial for detecting and discriminating external objects and events. The timing of auditory and visual events is especially important for appraising and navigating dynamically changing environments. Previous studies have demonstrated that sensory modality affects time perception. The perceived duration of a short tone was longer than that of a light of equal length that were presented separately^1^. Duration discrimination of short time intervals was better in the auditory mode than in the visual mode^2^. Similarly, temporal sensitivity was higher in the auditory mode than in the visual mode for various types of timing tasks^3^. Temporal discrimination of auditory and visual signals was worse than that of signals in the same sensory modality^4^. Mayer et al.^5^ supported the idea of a single modality-independent timekeeping mechanism that enables duration judgement for auditory, visual, and tactile stimuli^5^. However, the neuronal processing involved in multisensory integration for timing is not yet fully understood.

The prefrontal cortex (PFC) has widespread connectivity with various cortical areas. The PFC receives multiple sensory signals, mainly from sensory association areas, and influences many brain regions involved in motor and cognitive processes. Neuroimaging studies have shown that the neural systems associated with cognitively controlled time measurement depend on prefrontal and parietal cortical regions^6^. In nonhuman primates, the dorsolateral PFC was activated in temporal discrimination tasks, and bicuculline injections into this area impaired temporal discrimination^7^. Neurophysiological studies have demonstrated that PFC neurons are widely associated with temporal information processing. PFC neurons showed phasic activity depending on the preceding delay period in a saccade task^8^. In a duration discrimination task with visual stimuli, PFC neurons exhibited differential activity depending on whether the first or the second stimulus was longer after two stimulus presentations^9,10^, and showed phasic activity during presentation of the stimuli that may serve to temporally categorize the ongoing stimulus^11^. Neurophysiological studies also showed that PFC neurons responded to both auditory and visual stimuli. Single PFC neurons integrate auditory and visual stimuli for communication^12^, and such multisensory PFC neurons were shown to be sensitive to the temporal properties of auditory and visual stimuli^13^. Therefore, the PFC is one of the likely candidates that integrates multisensory signals for timing.

**Figure 1:**
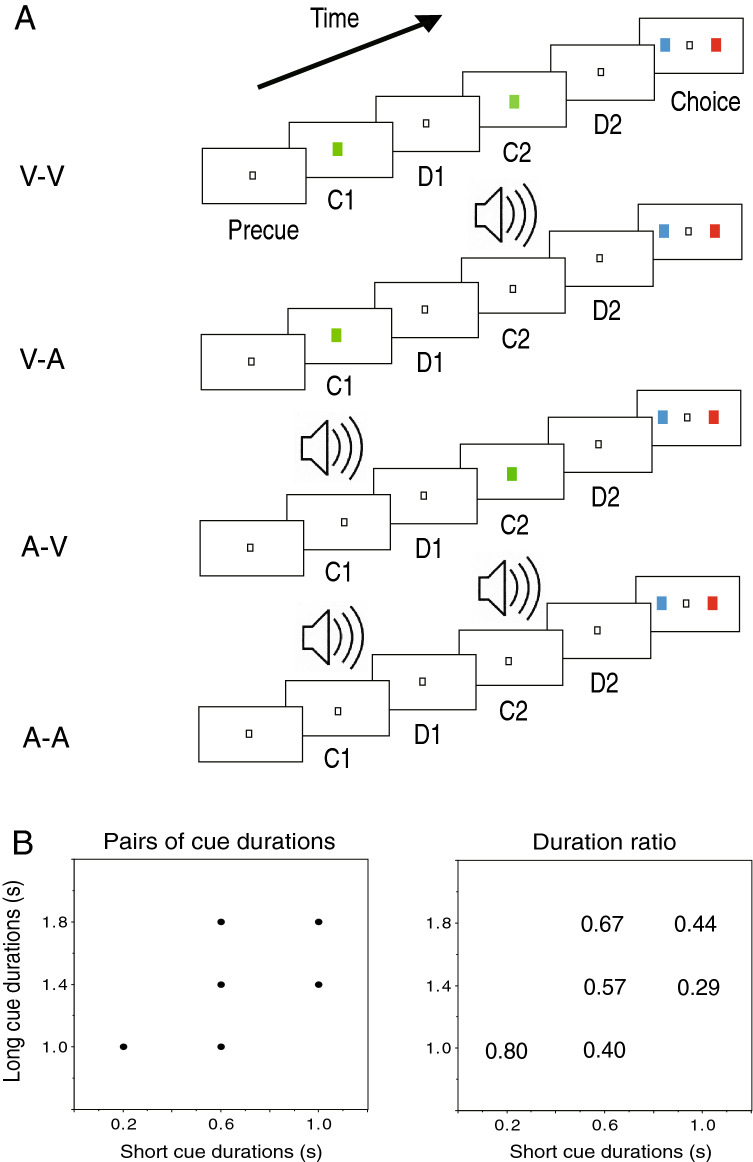
Behavioral task and pairs of cue durations. (**A**) Sequences of the duration discrimination task with auditory and visual cues. C1, first cue; D1, first delay; C2, second cue; D2, second delay. Four modality sequences of C1 and C2 were adopted: V-V, visual-visual; V-A, visual-auditory; A-V, auditory-visual; A-A, auditory-auditory. (**B**) Pairs of cue durations (left) and cue duration ratios (right) used in the task. Cue duration ratio = (long duration − short duration)/long duration.

In the present study, to investigate neuronal processing involved in the integration of auditory and visual signals for time perception, neuronal activity was examined in the PFC while monkeys were performing a duration discrimination task with auditory and visual stimuli (Fig. 1A). In particular, the following questions were addressed. Do PFC neurons show the same timing-related activity with auditory stimuli as they do with visual stimuli? If so, do single PFC neurons respond to both auditory and visual stimuli? In addition, do PFC neurons exhibit timing-related activity that integrates information about auditory and visual stimuli?

## Results

### Behavioral performance

**Figure 2:**
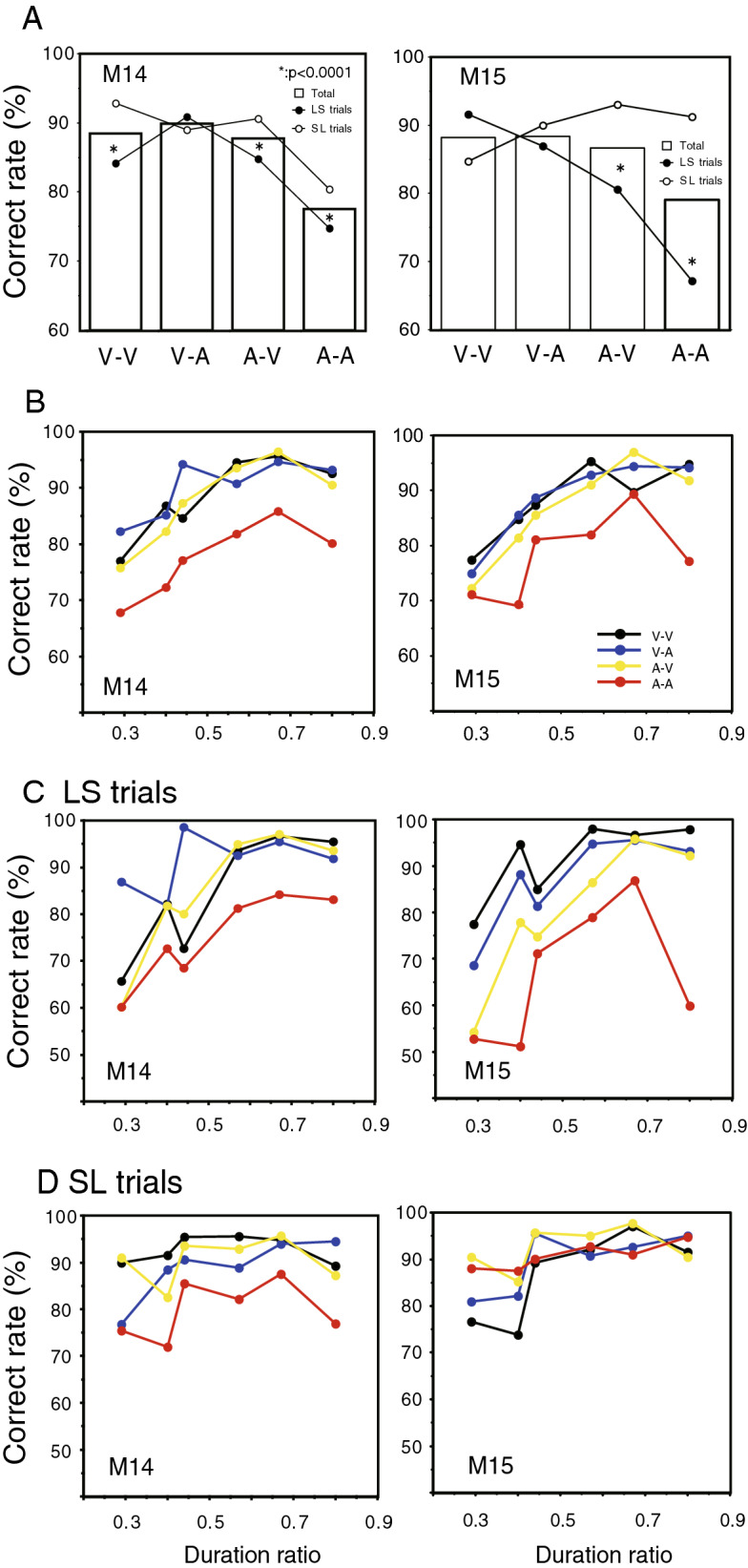
Correct rates for M14 (left column) and M15 (right column). (**A**) Correct response rates with the four cue modality sequences. Bar graphs show correct rates for each type of modality sequence. Line graphs show correct rates to the four modality sequences in the LS (long-short, black circle) and the SL (short-long, white circle) trials separately. Significant differences in correct rates between the LS and the SL trials are shown by asterisks (*p* < 0.0001). (**B**) Correct rates as a function of duration ratios. Correct rates are plotted against duration ratios using the same color symbols for each type of modality sequence. (**C**,**D**) Correct response rates as a function of duration ratios separately shown for the LS (**C**) and the SL (**D**) trials.

Figure 2A shows correct rates in each monkey to four cue modality sequences: visual-visual (V-V), visual-auditory (V-A), auditory-visual (A-V) and auditory-auditory (A-A). Data from a total of 19058 trials from M14 [9556 long-short (LS) trials and 9502 short-long (SL) trials) and 17418 trials from M15 (8773 LS trials and 8645 SL trials) were collected during stable performance sessions for this analysis. Correct rates were significantly different among the four modality sequences in both monkeys (ANOVA, *p* < 0.0001). Correct rates with the A-A sequences were lower than those with the other three sequences (bar graphs in Fig. 2A). Comparing correct rates between the LS and the SL trials in each modality sequence, those on the LS trials were significantly lower than those on the SL trials with the A-V and A-A sequences for both monkeys (Student’s t-test, *p* < 0.0001; line graphs in Fig. 2A). Correct rates were separately plotted against the duration ratio separately for the four modality sequences in each monkey in Fig. 2B. Correct rates increased as the duration ratio increased in the lower range of the duration ratio, and were relatively constant in the higher range for both monkeys, with the exception of the highest ratio on the A-A trials. The relationships between correct rates and the duration ratio across the four modality sequences are shown separately for the LS trials (Fig. 2C) and for the SL trials (Fig. 2D). Correct rates increased as the duration ratio increased more clearly on the LS trials than on the SL trials with all four modality sequences for both monkeys.

### Neuronal activity

A total of 860 neurons were recorded from both sides of PFC in the M14 (n = 539) and from the left side of the PFC in M15 (n = 321). Both monkeys used the right hand to perform the task. Therefore, the activity was recorded from the contralateral side of the performing arm in both monkeys and, in addition, from the ipsilateral side in M14. Out of the 860 neurons, 57, 102, 32 and 136 neurons were responsive to the first cue (C1), second cue (C2), first delay (D1), and second delay (D2), respectively.

### C1-responsive neurons

**Figure 3:**
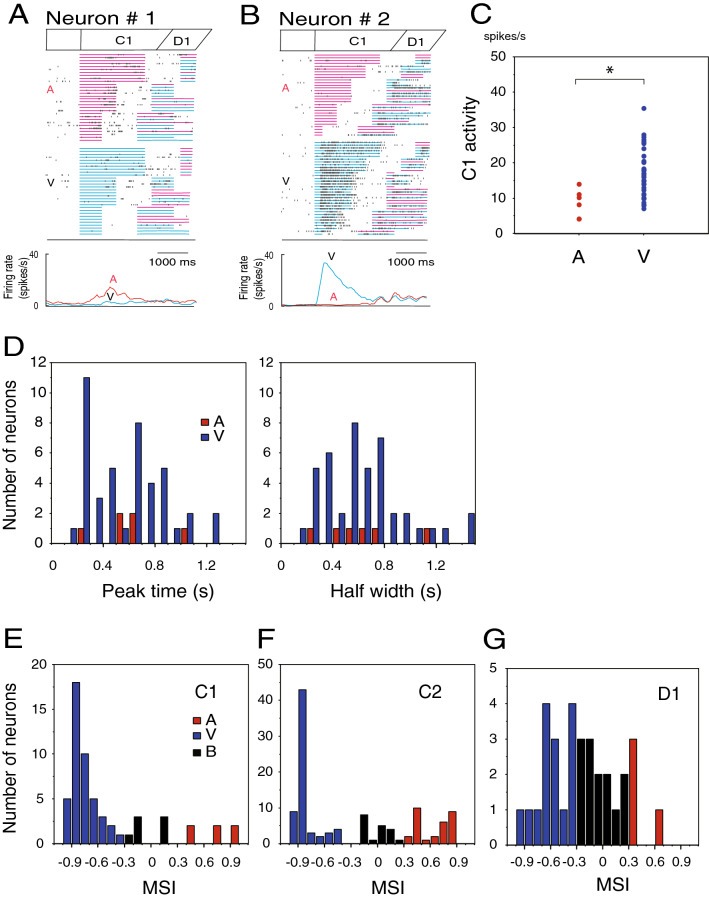
Activity of C1-responsive neurons. (**A**) An auditory C1-responsive neuron. A raster diagram and firing rate curves are aligned with C1 onset. In the raster display, which is separately shown in auditory (A) and visual (V) C1 trials and is rearranged by the C1 duration from long to short, a row corresponds to a trial. In each row, small black dots represent times of neuronal firing. Red and blue horizontal lines indicate auditory and visual cue presentations. For firing rate curves, firing rates were averaged in 200-ms sliding-time windows, moved in 40-ms steps, separately for auditory (red) and visual (blue) C1 trials. (**B**) A visual C1-responsive neuron. (**C**) Auditory and visual responses of the C1-responsive neurons. A scatter plot shows the mean firing rates of each auditory C1-responsive neuron (n = 6, red) during the auditory C1 period and those of each visual C1-responsive neuron (n = 43, blue) during the visual C1 period. A significant difference between the auditory and visual responses was detected (*p* < 0.05) (shown by *). (**D**) Peak times and half widths of C1 activity. Distributions of peak times from C1 onset (left) and half widths (right) are displayed for the auditory responses (red bars) of auditory C1-responsive neurons and the visual responses (blue bars) of visual C1-responsive neurons. (**E**) Distribution of the modality sensitivity index (MSI) for the C1-responsive neurons. MSI = (AUD − VIS)/(AUD + VIS). AUD is the mean firing rate during auditory C1 presentation and VIS is the mean firing rate during visual C1 presentation. A neuron was defined as auditory (red bars) when the MSI was greater than 0.3, and as visual (blue bars) when the MSI was less than − 0.3; otherwise, it was defined as bimodal (B, black bars). (**F**) Distribution of the MSI for the C2-responsive neurons. (**G**) Distribution of the MSI for the D1-responsive neurons.

C1-responsive neurons exhibited C1 modality-specific activity during the C1 period. Out of 57 C1-responsive neurons, 6 neurons responded only to an auditory C1, 44 neurons responded only to a visual C1, and 7 neurons responded to both auditory and visual cues (Table). The distribution of the modality sensitivity index (MSI) for the C1- responsive neurons is shown in Fig. 3E. Figure 3 shows examples of modality-specific C1-responsive neurons. The neuron in Fig. 3B exhibited vigorous phasic activity only during the visual C1 period with a peak time of 0.2 s from C1 onset, while the neuron in Fig. 3A showed weak activity only during the auditory C1 period with a peak time of 0.8 s. Figure 3C shows the mean firing rates during the C1 period for the visual and auditory C1-responsive neurons. The visually responsive neurons showed greater C1 activity than the auditory responsive neurons. Figure 3D shows distributions of peak times and half widths of C1 activity to the visual and auditory C1. The visual responses tended to peak earlier from the C1 onset than the auditory responses. These results suggested that the temporal durations of auditory and visual C1 might be separately processed in the PFC. Half widths of C1 responses did not differ between the auditory and the visual responses.

**Table 1:** Cue modality sensitivity of C1- and C2-responsive neurons.

	Auditory	Visual	Bimodal	Total
C1	6 (11%)	44 (77%)	7 (12%)	57 (100%)
C2	22 (23%)	64 (62%)	16 (15%)	102 (100%)
Both C1 & C2	0 (0%)	25 (96%)	1 (4%)	26 (100%)

### C2-responsive neurons

**Figure 4:**
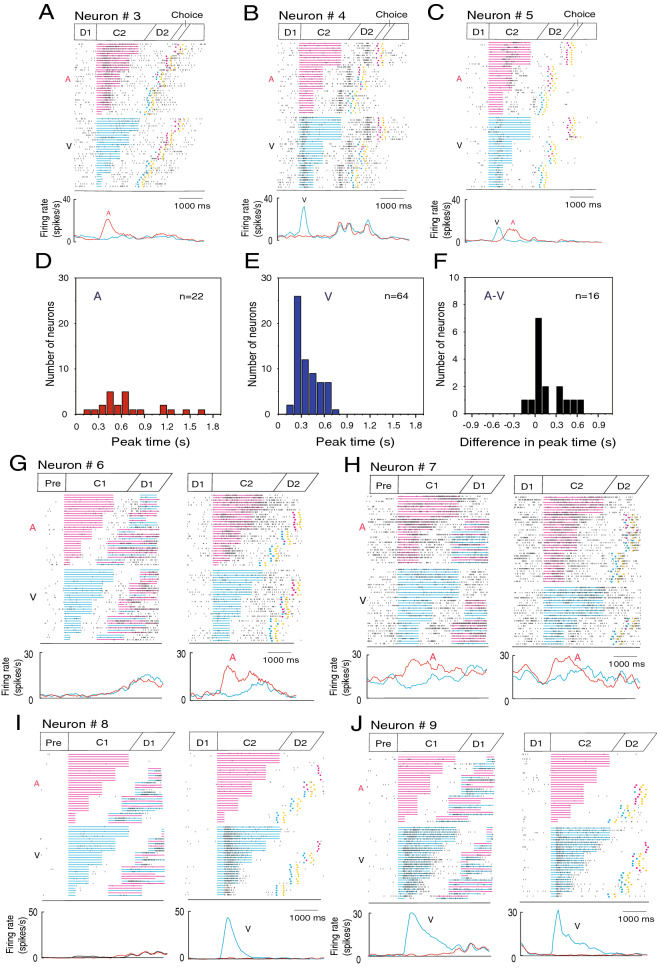
Activity of C2-responsive neurons. (**A**) An auditory C2-responsive neuron. A raster diagram and firing rate curves are aligned with C2 onset. The raster display is separately shown in auditory (A) and visual (V) C2 trials and is rearranged by the C2 duration from long to short. Red and blue squares represent times of red and blue button presses, and yellow squares represent times of reward delivery. Firing rate curves show changes in activity for the auditory (A, red) and visual (V, black) C2 trials. (**B**) A visual C2-responsive neuron. (**C**) A bimodal C2-responsive neuron. (**D**) Distributions of peak times from C2 onset of auditory C2 responses for auditory C2- responsive neurons. (**E**) Distributions of peak times of visual C2 responses for visual C2-responsive neurons. (**F**) Distributions of differences in peak time between visual and auditory C2 responses for bimodal C2-responsive neurons. Difference in peak time = (peak time of auditory responses) − (peak time of visual responses). (**G**) An auditory C2-responsive neuron without any C1 response. *Left:* A raster diagram and firing rate curves are aligned with C1 onset for C1 responses. The raster display is rearranged by the C1 duration from long to short. Firing rate curves show changes in activity for the auditory (A, red) and visual (V, blue) C1 trials. *Right:* A raster diagram and firing rate curves are aligned with C2 onset for C2 responses. The raster display is rearranged by the C2 duration from long to short. Firing rate curves show changes in activity for the auditory and visual C2 trials. (**H**) An auditory C2-responsive neuron with auditory C1 responses. (**I**) A visual C2-responsive neuron without any C1 response. (**J**) A visual C2-responsive neuron with visual C1 responses.

C2-responsive neurons exhibited C2 modality-specific activity during the C2 period. Out of 102 C2-responsive neurons, 22 neurons responded only to an auditory C2, 64 neurons responded only to a visual C2, and 16 neurons responded to both auditory and visual cues (Table 1). The number of C2-responsive neurons was almost twice as many as that of C1-responsive neurons. The distribution of the MSI for the C2-responsive neurons is shown in Fig. 3F. Figure 4 shows examples of modality-specific C2-responsive neurons. The neuron in Fig. 4A showed phasic activity only during the auditory C2 period with a peak time of 0.6 s, while the neuron in Fig. 4B exhibited vigorous phasic activity only during the visual C2 period with an early peak time of 0.2 s from the C2 onset. The bimodal neuron in Fig. 4C exhibited visual C2 activity with a peak time of 0.5 s and the auditory C2 activity with a peak time of 1.0 s.

Figure 4D,E show distributions of peak times of C2 responses for the auditory and visual C2-responsive neurons. Visual C2 responses showed earlier peak times than auditory responses. For the bimodal C2-responsive neurons, Fig. 4F demonstrates differences in peak time between visual and auditory C2 responses. Visual responses also tended to peak earlier from the C2 onset than auditory responses in the bimodal neurons. Phasic cue activity may have a filtering role based on peak times in attempted duration discrimination^11^. Differences in peak times between visual and auditory cue activity might result in differences in duration detection between visual and the auditory cues.

The majority of C2-responsive neurons responded only to C2 but not to C1 (Table 1). The C2-responsive neuron in Fig. 4G responded only to auditory C2 but not to auditory C1. Similarly, the C2-responsive neuron in Fig. 4I responded only to the visual C2 but not to the visual C1. The majority of C1-responsive neurons also responded only to C1 but not to the C2. These task period-dependent cue responses would not be simple sensory responses since simple responses could have been elicited similarly by C1 and C2. A small group of cue-responsive neurons responded to both C1 and C2 (Table 1). In these dual response neurons, the specificity to the cue modality did not change between C1 and C2. The cue-responsive neuron in Fig. 4H responded to auditory C1 and C2, while the neuron in Fig. 4J responded to visual C1 and C2.

### D1-responsive neurons

**Figure 5:**
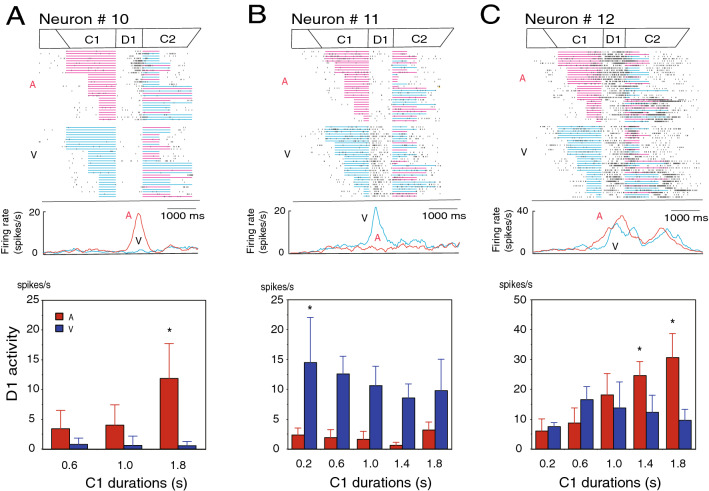
Activity of D1-responsive neurons. (**A**) A D1-responsive neuron exhibiting D1 activity only after auditory C1. A raster diagram and firing rate curves are aligned with D1 onset. The raster display is separately shown in auditory (A) and visual (V) C1 trials and is rearranged by the C1 duration from long to short. Firing rate curves show changes in activity for the auditory (A, red) and visual (V, blue) C1 trials. A bar graph (bottom) shows mean firing rates during the D1 period to each C1 duration in the auditory (red) and visual (blue) C1 trials for this neuron. The activity in the trials with a C1 duration of 1.8-s was greater than that in other types of trials (shown by *). (**B**) A D1-responsive neuron showing D1 activity only after visual C1. The activity in the trials with a C1 duration of 0.2-s was greater than that in other types of trials (shown by *). (**C**) A D1-responsive neuron showing D1 activity following auditory and the visual C1. The activity in the trials with auditory C1 durations of 1.4- and 1.8-s was greater than that in other types of trials (shown by *).

D1-responsive neurons showed C1 modality-specific activity during the D1 period. Out of 32 D1 response neurons, 4 neurons exhibited greater D1 activity after auditory C1 than visual C1, while 15 neurons showed larger D1 activity after visual C1 than auditory C1. The distribution of the MSI for the D1-responsive neurons is shown in Fig. 3G. Furthermore, 12 neurons showed the activity that changed depending on C1 duration (Table 2). This number might be just what would be expected by chance. Therefore, we addressed this possibility using a bootstrap method to assess the result. A bootstrapping analysis for the probability with 10,000 samples yielded a 95% confidence interval of 3.876–15.85 neurons. This result means that the number of neurons was significant and was not induced by chance, although it was a small number of neurons. The D1-responsive neuron in Fig. 5A exhibited D1 activity following an auditory C1 of a 1.8-s duration but not a 0.6- and 1.0-s duration. The D1-responsive neuron in Fig. 5B showed marked D1 activity after visual C1. This D1 activity changed depending on C1 duration (ANOVA; F (4, 61) = 3.22, *p* < 0.025). The activity in the trials with the C1 of 0.2 s was greater than that in the other trials (Tukey–Kramer test, *p* < 0.05). The D1-responsive neuron in Fig. 5C showed marked D1 activity after auditory C1. This D1 activity changed depending on C1 duration (ANOVA; F (4, 62) = 30.75, *p* < 0.0001). The activity in the trials of a C1 with a 1.8- and 1.4-s duration was greater than that in the other trials (Tukey–Kramer test, *p* < 0.05). D1 activity represented temporal information concerning C1 duration, and the activity representing C1 duration was affected by C1 modality.

**Table 2:** C1 modality sensitivity and C1 duration dependency in D1-responsive neurons.

C1 modality	C1 duration dependency		Total
	Dependent	Independent	
Auditory	3 (75%)	1 (25%)	4 (100%)
Visual	4 (27%)	11 (73%)	15 (100%)
Bimodal	5 (38%)	8 (62%)	13 (100%)

### D2-responsive neurons

**Figure 6:**
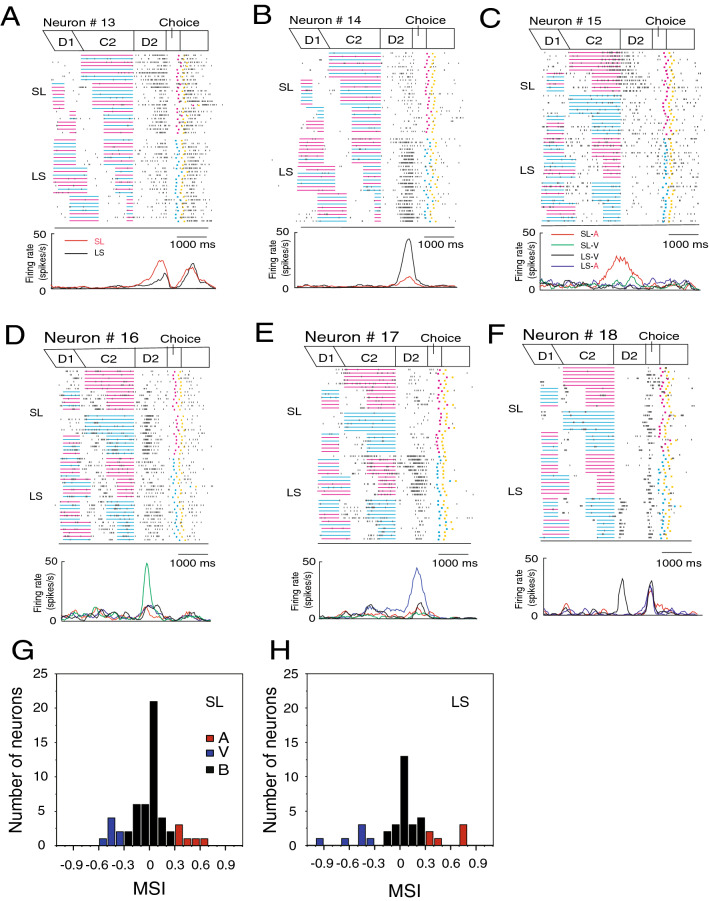
Activity of D2-responsive neurons. (**A**) A D2-responsive neuron showing greater D2 activity in the SL trials than in the LS trials. A raster diagram and firing rate curves are aligned with D2 onset. The raster display is separately shown in the SL and LS trials and is rearranged by the C2 duration from long to short. Firing rate curves show changes in activity for the SL (red) and LS (black) trials. (**B**) A D2-responsive neuron showing greater D2 activity in the LS trials than in the SL trials. (**C**) A D2-responsive neuron showing selective D2 activity in the SL trials with auditory C2. The raster display is separately shown in the SL and LS trials and for the C2 modality. Firing rate curves show changes in activity for the SL trials with auditory (red) and visual (green) C2, and in the LS trials with auditory (blue) and visual (black) C2. (**D**) A D2-responsive neuron showing selective D2 activity in the SL trials with visual C2. (**E**) A D2-responsive neuron showing selective D2 activity in the LS trials with auditory C2. (**F**) A D2-responsive neuron showing selective D2 activity in the LS trials with visual C2. (**G**) Distribution of the MSI to the LS type of D2-responsive neurons. MSI = (VIS − AUD)/(AUD + VIS). AUD is D2 activity after auditory C2, and VIS is the D2 activity following visual C2. (**H**) Distribution of the MSI to the SL type of D2-responsive neurons.

D2-responsive neurons exhibited duration order-sensitive activity during the D2 period. The activity changed depending on whether C1 or C2 had been presented longer. Out of 136 D2-responsive neurons, 54 neurons showed greater D2 activity in the SL trials than in the LS trials (SL type; Table 3). In the SL trials, C2 was presented longer than C1. On the other hand, 37 neurons exhibited higher D2 activity in the LS trials than in the SL trials (LS type; Table 3). In the LS trials, C1 was presented longer than C2. Figure 6 shows examples of such duration order-sensitive D2-responsive neurons. The D2-responsive neuron in Fig. 6A showed greater D2 activity in the SL trials than in the LS trials, while the neuron in Fig. 6B exhibited higher D2 activity in the LS trials than in the SL trials.

**Table 3:** Duration order specificity and C2 modality sensitivity in D2-responsive neurons.

Duration order	C2 modality			Total
	Auditory	Visual	Bimodal	
SL type	6 (11%)	7 (13%)	41 (76%)	54 (100%)
LS type	6 (16%)	6 (16%)	25 (68%)	37 (100%)

Nearly one third of the duration order-sensitive D2-responsive neurons demonstrated the C2 modality-specific activity. The neuron in Fig. 6C showed D2 activity in the SL trials only when C2 was auditory, while the neuron in Fig. 6D exhibited D2 activity in the SL trials only when C2 was visual. The neuron in Fig. 6E showed D2 activity in the LS trials only when C2 was auditory, while the neuron in Fig. 6F exhibited D2 activity in the LS trials only when C2 was visual. Duration order sensitivity and C2 modality specificity in D2-responsive neurons are summarized in Table 3. The distribution of the MSI for the LS and the SL types of D2-responsive neurons is shown in Fig. 6G,H. D2 activity represented order-based duration, that is, whether C1 or C2 was longer, and this activity was partly affected by C2 modality.

### Recording sites

**Figure 7:**
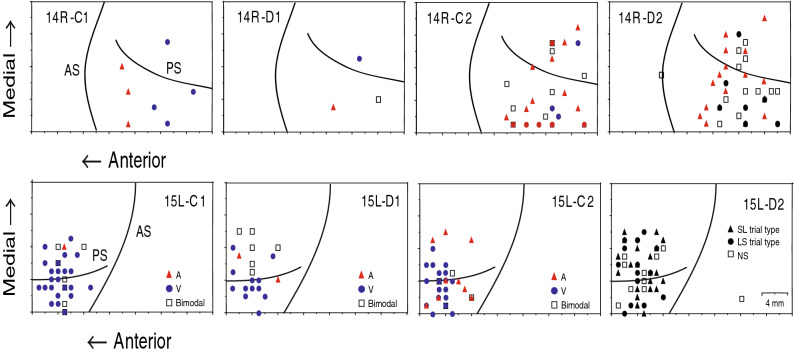
Recording sites. Cortical surface maps show penetration sites where C1-, D1-, C2-, and D2-responsive neurons were recorded in the right PFC of monkey M14 (top) and in the left PFC of monkey M15 (bottom). In the maps for C1-, D1- and C2-responsive neurons, symbols represent the sites where neurons responding to auditory (red triangles), visual (blue circles), and both auditory and visual (white squares) cues were found. In the maps for D2- responsive neurons, symbols indicate the sites where neurons showing selective D2 activity in the SL (black triangles) and LS (black circles) trials and nonselective D2 activity (white squares) were recorded. AS, arcuate sulcus; PS, principal sulcus.

Figure 7 illustrates penetration sites where the C1-, D1-, C2-, and D2-responsive neurons were recorded in the right PFC of M14 (upper panels) and in the left PFC of M15 (lower panels). Most neurons were recorded within 3 mm from the cortical surface at each penetration site. Differences in cortical distribution were not distinguished among the C1-, D1-, C2-, and D2-responsive neurons. Visual and bimodal C1- and C2-responsive neurons were found in the dorsolateral PFC without spatial preference, although the proportion and distributions of auditory responsive neurons differed between monkeys and hemispheres. The SL and the LS types of D2-responsive neurons were also recorded in the dorsolateral PFC without spatial preference.

## Discussion

The present study examined neuronal activity in the PFC during the duration discrimination task with auditory and visual cues in order to clarify neuronal processing involved in the integration of auditory and visual signals for time perception. PFC neurons showed timing-related activity in response to auditory cues and visual cues. Although a small number of single PFC neurons responded to both auditory and visual cues, the majority of the cue-responsive neurons were modality specific. After sequential presentation of auditory and visual cues, PFC neurons exhibited phasic activity representing which cue, the first or the second cue, had been presented longer. This activity representing order-based duration was partly affected by the cue modality. These results suggested that temporal information on visual and auditory signals might be separately processed in the PFC in the early stage of duration discrimination, and integrated for the final decision in the late stage.

Regarding the behavioral performance, correct response rates were affected by cue modality sequences and cue duration order in the present study. Correct rates with the A-A sequences were lower than those with the other three sequences, i.e., the V-V, V-A, and A-V sequences. Better performance was observed in the SL trials than in the LS trials, especially with the A-V and A-A sequences, in which auditory C1 was longer than the C2. These results do not necessarily agree with previous reports on human psychological experiments.

Grondin et al.^2^ showed that discrimination performance was better in the auditory mode than in the visual mode in duration discrimination with a range of sub-second stimuli. Temporal sensitivity was markedly higher for auditory than for visually presented intervals in duration discrimination with base durations below 600 ms^14^. The auditory modality tends to dominate over the visual modality in bimodal temporal perception^15^. In temporal discrimination using visual and auditory stimuli, better performance was observed in the congruent trials in which the two stimuli were of the same modality than in the incongruent trials in which the two stimuli were of different modalities^4^. In the present study, A-A and V-V were congruent trials, and V-A and A-V were incongruent trials. Previous psychological experiments have shown that durations of visual stimuli were classified as shorter than equivalent durations signaled by auditory stimuli in a duration bisection task^16,17^. Moreover, subjective duration is affected by nontemporal stimulus properties, the allocation of processing resources, and past experience with the stimulus^18^.

Two general frameworks have been articulated to describe how temporal duration is perceived. One is a dedicated neural mechanism specialized for representing temporal duration, and the other is an intrinsic dynamic mechanism through the activation of sensory processes^19^. The differences in performance between modalities and among the task sequences, which were observed in the present experiment, might be consistent with state-dependent temporal computations^20^, and in line with the framework of intrinsic, modality-specific neural mechanisms for interval timing.

PFC neurons showed phasic activity during the C1 and the C2 periods, when the subjects perceived the duration of auditory and visual cues, in the present study. The majority of the cue-responsive neurons responded to either the auditory cue or the visual cue. The dorsolateral area of the PFC receives projections from the auditory association area in addition to the visual association areas^21^. Auditory responses and visual responses have been reported in this area. A group of PFC neurons, which were different from visually responsive neurons, responded to a sound to obtain information on the contralateral space around the animal^22^. In a delayed matching-to-sample task, separate groups of PFC neurons were activated with respect to the location of visual or auditory stimuli, suggesting that working memory regarding auditory and visual space involves modality-specific mechanisms^23^. PFC neurons were also shown to encode the task phase indicated by visual, auditory, and tactile sensory signals^24^.

Cue responses in the present study were unlikely to be simple sensory responses to the auditory stimulus or visual stimulus because the majority of the cue-responsive neurons responded to either C1 or C2. If the responses were simple sensory responses, the neurons would have responded to both C1 and C2. Cue responses could be involved in interval timing of auditory and visual stimuli, although the auditory and visual responses during the C1 and C2 periods might be sensory responses not related to timing computations. Oshio et al.^11^ demonstrated that phasic activity with constant delay from the cue onset could function to filter current cue duration based on peak time. The C1- and C2-responsive neurons might separately contribute to timing cue duration with temporal filtering for the visual and auditory cues. In the C1- and the C2-responsive neurons, visual responses tended to peak earlier relative to cue onset than auditory responses, and this pattern was observed even in a small number of bimodal response neurons. Differences in peak time between the visual and the auditory cue responses might have resulted in differences in duration detection between the visual and auditory cues and contributed to differences in correct performance rates among the cue modality sequences.

In the present study, PFC neurons showed phasic activity during the D1 period, when the subjects needed to keep the stimulus duration in memory. However, only a small number of D1-responsive neurons showed activity that changed depending on the C1 duration. Therefore, we have not clarified how PFC neurons code C1 duration in the present study. Oshio et al.^9^ demonstrated that PFC neurons exhibited differential activity following a short or long cue in a duration discrimination task with visual stimuli, suggesting that PFC neurons represent categorical duration of visual cues, i.e., long or short, for the subsequent duration discrimination of visual stimuli. Genovesio et al.^8^ reported that the phasic activity of PFC neurons changed depending on the duration of the preceding visual stimulus. Furthermore, PFC neurons have been shown to encode not only relative but also absolute durations of visual stimuli in a duration discrimination task^10,25^. The present study does not contradict these results. Moreover, PFC neurons encoded the duration of the preceding auditory stimulus as well as a visual stimulus.

In the present study, PFC neurons showed phasic activity during the D2 period, when the subjects decided whether the presentation of C1 or C2 had been longer and retained the comparison result. D2 activity was different between the LS and SL trials, representing order-based duration, that is, whether the C1 or C2 was presented for a longer period of time. Oshio et al.^9^ demonstrated that PFC neurons exhibited the same type of activity representing order-based duration in a duration discrimination task with visual stimuli. Similar activity was also reported in the PFC by Genovesio et al.^10^ and in the striatum by Chiba et al.^26,27^. The present study confirmed these previous reports. However, in the present study, the D2-responsive neurons representing order-based duration could alternatively or conjunctively represent the target color associated with the relative cue duration. We do not rule out this possibility because we have not attempted to reverse the color rule in this task.

Moreover, PFC neurons encoded order-based durations of auditory stimuli as well as visual stimuli. Romanski and Hwang reported that PFC neurons integrated auditory and visual information and were sensitive to the timing of visual and auditory stimuli for the integration of multisensory communication information^13^. A group of PFC neurons in the present study showed the comparable D2 activity among four modality sequences, while other groups of neurons exhibited D2 activity affected by C2 modalities. These results suggested that the PFC may process duration information on visual and auditory stimuli for duration discrimination, partially in parallel for different modalities, and integrate them for the final decision. A group of D2-responsive neurons showed differential activity between the LS and the SL trials, regardless of cue modalities. This PFC activity could represent the final decision in duration discrimination.

Interval timing depends on the interaction between the core timing structures and context-dependent areas^28^. The dorsolateral PFC is essential for cognitively controlled time measurement^29^ and for the storage and recovery of memory of a given time period^30^. In neuroimaging studies, timing tasks, especially cognitively controlled timing tasks, frequently show activation in the dorsolateral PFC^31^. A patient with ischemic lesion in the right dorsolateral PFC had trouble estimating the duration of events^32^. Stroke patients with PFC lesions exhibited a significant timing deficit in a time discrimination task^33^. Healthy subjects underestimated time periods in a time reproduction task after inhibitory repetitive transcranial magnetic stimulation (TMS) over the dorsolateral PFC^30^. PFC neurons exhibited phasic activity representing stimulus duration after presentation of a visual stimulus^9,26^, and coded relative cue duration after presentation of two visual cues^9,10^.

The PFC is connected with auditory, visual, and somatosensory association cortical areas and with multimodal cortical areas^34^. The PFC receives information regarding auditory stimuli as well as visual stimuli from these areas and integrates relevant information. PFC neurons responded during a delay period following auditory cue presentation in a delayed matching-to-sample task^23^. Ventrolateral PFC neurons responded to both faces and vocalizations, and these multisensory neurons were sensitive to the temporal properties of the audiovisual stimuli^13,35^. In a neuroimaging study PFC activation was elicited by simultaneity judgements and temporal order judgements with nonsemantic auditory and visual stimuli^36^. In the present study, PFC neurons exhibited the activity representing the order-based duration of auditory and visual cues in the duration discrimination task. The PFC is likely to integrate temporal information related to auditory and visual stimuli for duration discrimination.

## Methods

Two male Japanese monkeys (*Macaca fuscata*, M14 and M15), weighing 8.0 kg and 9.2 kg, were used in the present study. The animals were provided through National BioResource Project “Japanese Macaques” of MEXT of Japan. All animal care and experimental procedures were approved by the Animal Care and Use Committee of Kindai University (KAME-19-005, KAME-26-023), and complied with the Guidelines for Proper Conduct of Animal Experiments of the Science Council of Japan (2006) and the institutional guidelines for the care and use of nonhuman primates. Additionally, this study was carried out in compliance with the ARRIVE guidelines. The animals were housed in the institutional animal center under daily supervision of veterinary staff, and were acclimatized to the experimental environment while sitting in custom-fitted primate chairs. Water intake was regularly controlled such that the monkeys were motivated to perform a behavioral task.

### Behavioral task

The monkeys were trained to perform a duration discrimination task with visual and auditory cues. The experimental apparatus was almost the same as that previously described^9,27^. The subjects sat in a primate chair in a dimly lit, electrically shielded room, and faced a flat panel equipped with a 6.5-inch display and three buttons under the display. All visual stimuli were presented on the display against a black background. Each trial was initiated when the subjects pressed the center hold button with the right hand, making a small white spot (2° × 2°) appear for 1.0 s at the center of the display. This period was referred to as the precue period. After the precue period, two cues of different durations ranging from 0.2 to 1.8 s were successively presented. Each of the two cues was followed by a delay period of 1.0 s during which the small white spot was displayed. The first cue, first delay, second cue, and second delay are referred to as C1, D1, C2, and D2, respectively. After the D2 period, blue and red squares (8° × 8° for each) were simultaneously presented on the left and right sides on the display, indicating the start of a choice period. Squares with blue and red colors were randomly presented on the two sides of the screen. The monkeys were trained to press the button below the blue square when C1 was longer than C2, and to press the button below the red square when C2 was longer than C1. The button press had to occur within 1.5 s after the start of the choice period. Correct responses were rewarded with a drop of juice. The subjects needed to keep pressing the hold button from the start of a trial until the onset of the choice period. If the subjects released the hold button before the choice period or took longer than 1.5 s to press the response button during the choice period, a stimulus was turned off and the trial was aborted. No extra feedback signal was given for incorrect responses. The monkeys spontaneously chose the time when to start each trial after an intertrial interval of 2.0 s.

Cues were either visual or auditory. The visual cue was a green square (8° × 8°) presented at the center of the display. The auditory cue was a 2000-Hz tone from a speaker in the front of the subjects. A small white spot remained on the display during auditory cue presentation. Four modality sequences of the C1 and the C2 were adopted (Fig. 1A): visual-visual (V-V), visual-auditory (V-A), auditory-visual (A-V) and auditory-auditory (A-A). For the short cue, durations of 0.2, 0.6 and 1.0 s were adopted, and for the long cue, durations of 1.0, 1.4 and 1.8 s were used. Figure 1B shows the pairs of cue durations adopted in the present task and the cue duration ratios ((long duration – short duration) / long duration) for each pair of cue durations. For each duration pair, two types of duration orders [long–short (LS) or short–long (SL)] and four modality sequences (V-V, V-A, A-V, and A-A) could be adopted. In each trial, a duration pair, duration order, a modality sequence, and a correct target position (left or right) were pseudorandomly determined. The task was controlled using the TEMPO system (Reflective Computing, St. Louis, USA). The behavior of the monkeys during task performance was monitored through a CCD camera placed on a ceiling in the shielded room.

### Surgical procedures and single-unit recordings

Surgical procedures and neurophysiological data collection were almost the same as those previously described^9,26^. After the monkeys demonstrated asymptotic performance in the task, they underwent aseptic surgery to implant recording chambers and two stainless steel pipes for head fixation on the skull. General anesthesia was initially induced using ketamine hydrochloride (5 mg/kg, i.m.) and xylazine hydrochloride (2 mg/kg, i.m.) with atropine sulfate (0.01 mg/kg, i.m.), and maintained with sodium pentobarbital (15 mg/kg, i.v., once every hour). Postoperatively, the subjects were given antibiotics (cefotiam hydrochloride, 10 mg/kg, i.m.) twice a day for one week to minimize the possibility of infection and an analgesic (meloxicam, 0.2 mg/kg, i.m.) once a day for 3 days. Daily recording sessions started after full recovery from the surgery.

Single-unit activity was recorded with epoxy-insulated tungsten microelectrodes (1–2.5 MΩ at 1 kHz; FHC, Bowdoinham, USA). The electrode was advanced into the PFC (45° from vertical in the frontal plane) through the dura matter with a hydraulic microdrive manipulator (MO-951C, Narishige, Tokyo, Japan) while the subjects were performing the task.

After filtering (bandpass: 150–3000 Hz) and amplification, spikes from a single neuron were isolated using the Multi-Spike Detector (MSD; Alpha Omega Engineering, Nazareth, Israel), which isolated the spiking activity based on an eight-point template-matching algorithm. Spikes and event data were saved with millisecond resolution by personal computers using the TEMPO system. The electrode was advanced in small steps while monitoring the signal waveform from the electrode on an oscilloscope and transmitting sound on a speaker. If a spike was accepted by the MSD, an accepted signal was also displayed with the corresponding spike on the oscilloscope for confirmation. The spike data for almost all well-isolated neurons were stored for off-line analysis. Activity was recorded from both sides of the dorsolateral PFC in M14 and the left side in M15.

### Data analysis

Data from correct trials were submitted for statistical analysis. First, in each neuron, mean firing rates were calculated for the precue, C1, C2, D1, and D2 periods. Then the C1, C2, D1, and D2 activities were compared with the baseline precue activity using the Mann–Whitney *U* test (significance level, *p* < 0.01) in each neuron. When a statistically significant difference was detected in these comparisons, the neuron was defined as a C1-, C2-, D1-, or D2-responsive neuron.

For the C1-responsive neurons, a modality sensitivity index (MSI) was calculated to characterize the C1 activity as auditory, visual, or bimodal responsive. MSI = (AUU − VIS)/(AUD + VIS). AUD is the mean firing rate during auditory C1 presentation and VIS is the mean firing rate during visual C1. We defined the activity as auditory when the MSI was greater than 0.3, as visual when the MSI was less than -0.3, and as bimodal otherwise. A similar analysis was performed for the C2-, D1- and D2-responsive neurons to define modality sensitivity. For C2-responsive neurons, AUD is the activity during auditory C2, and VIS is the activity during visual C2. In the case of the D1- and D2-responsive neurons, AUD is the activity following the auditory C1 or C2 period, and VIS is the activity after visual C1 or C2 presentation, respectively. Furthermore, Gaussian fitting analysis was performed for the C1-responsive neurons to characterize the phasic response by peak time and peak width. The details of this analysis were explained in our previous paper^9^.

For the D1-responsive neurons, the relationship between D1 activity and C1 duration was also examined. D1 activities following each C1 duration were compared with one-way ANOVA (significance level, *p* < 0.05). When a statistically significant difference was detected in the comparison, the neuron was considered as C1 duration dependent.

For the D2-responsive neurons, mean firing rates during the D2 period in the LS and the SL trials were compared with the Mann–Whitney *U* test (significance level, *p* < 0.05). When a statistically significant difference was detected in this comparison, the neuron was considered as an LS or an SL type of D2-responsive neuron. LS neurons showed greater D2 activity in the LS trials, and SL neurons exhibited greater D2 activity in the SL trials.

### Histology

After collecting neuronal data, small marking lesions were placed at known electrode coordinates by passing negative DC currents through the tips of microelectrodes (30 μA for 30 s). The monkeys were deeply anaesthetized with an overdose of sodium pentobarbital (60 mg/kg, intraperitoneal) and perfused transcardially with saline, followed by 10% formalin. The brain was immediately removed from the skull, and saturated with 10% sucrose and 30% sucrose at 4 °C. The preserved brain was cut serially into 50 μm-thick coronal sections on a freezing microtome. Every fourth section was histochemically stained using cresyl violet. Recording sites were reconstructed with reference to the marking lesions.
